# Modeling *Streptococcus pyogenes* Pharyngeal Colonization in the Mouse

**DOI:** 10.3389/fcimb.2019.00137

**Published:** 2019-05-02

**Authors:** Artemis Gogos, Michael J. Federle

**Affiliations:** ^1^Department of Microbiology and Immunology, University of Illinois at Chicago, Chicago, IL, United States; ^2^Department of Medicinal Chemistry and Pharmacognosy, Center for Biomolecular Sciences, University of Illinois at Chicago, Chicago, IL, United States

**Keywords:** pharyngeal colonization, pharyngitis, carriage, colonization model, innate immune response, host pathogen interaction

## Abstract

*Streptococcus pyogenes*, or Group A Streptococcus (GAS), is a human-restricted pathogen most commonly found in the posterior oropharynx of the human host. The bacterium is responsible for 600 million annual cases of pharyngitis globally and has been found to asymptomatically colonize the pharynxes of 4–30% of the population. As such, many studies have utilized animals as models in order to decipher bacterial and host elements that contribute to the bacterial-pharyngeal interaction and determine differences between acute infection and asymptomatic colonization. The aim of this review is to first describe both bacterial and host factors that are important for the pharyngeal persistence of GAS in humans, then to detail the bacterial and host factors that are important for colonization in murine model, and finally to compare the two in order to evaluate the strength of murine pharyngeal colonization as a model for the human-GAS pharyngeal interaction.

## Introduction

*Streptococcus pyogenes*, or Group A Streptococcus (GAS), is most commonly found in the posterior pharynx. Although GAS is restricted to human hosts, researchers have developed animal models to help decipher elements of both the host and bacterial defenses that play an important role in habitation of the pharyngeal mucosa. The most successful models have examined pharyngeal colonization in various non-human primates including baboons, rhesus macaques, chimpanzees, and the cynomolgus macaque (Watson et al., 1946; Friou, 1950; Taranta et al., 1969; Ashbaugh et al., 2000; Gryllos et al., 2001; Virtaneva et al., 2005). Non-human primates develop symptomatic infections similar to humans including pharyngeal erythema, fever, enlargement of the tonsils, and palatal petechiae following intranasal infection with GAS (Friou, 1950; Virtaneva et al., 2005). Unsurprisingly, the non-human primate model has emerged as the gold standard for oropharyngeal colonization models of GAS. Thus, far, it is the only model where symptoms similar to human pharyngitis can be observed and an immune response most like that of humans can be examined. However, the model is very expensive and technically challenging, and for these reasons has only been utilized a handful of times. Far more often, researchers will utilize mice as a cost-effective, accessible model in which to study the interaction between GAS and the host but the symptomatic pathology of human pharyngitis is not seen during nasopharyngeal infection with GAS in mice. In this review, we aim to delve into data resulting from these mouse studies to evaluate the model's validity as an approximation of the interaction between GAS and the human host.

In addition to evaluating the model's validity, we also hope to compile the first comprehensive index of data resulting from murine pharyngeal studies in the GAS literature. Oftentimes, mouse studies describe the relationship between the bacteria and pharyngeal tissues using various terms that have been inconsistently defined, including *colonization, carriage*, or *infection*. This variation in terminology makes it difficult to survey the GAS literature for instances using this model. Additionally, the terms themselves can be confusing. For example, some groups define *colonization* as a lack of clinical symptoms, while others define it as simply persistence of the bacterium for a certain period regardless of inflammatory or pathologic outcomes. For the purposes of this review, the term *colonization* will simply be defined as persistent inhabitation of the pharynx.

This review aims to carefully examine and present the data that is known regarding the molecular processes occurring between GAS and the human and compare it to data resulting from mouse models of pharyngeal colonization in order to evaluate the strength of the murine pharyngeal colonization as a model for the human-GAS pharyngeal interaction. Information on additional animal models of GAS infection, beyond murine respiratory tract infections, can be found in an excellent book chapter that reviews this topic (Watson et al., 2016).

## Characteristics of Pharyngeal Colonization in the Human Host

To accurately evaluate the translational power of pharyngeal GAS animal models, it is important to understand first the behavior of GAS in its native environment—the human oropharynx. GAS is considered a human-restricted bacterium, and thus it is undeniable that animal models will lack certain components of the native system. Furthermore, GAS has been shown to rely upon a number of human-specific factors in order to carry out its lifestyle in the host. A brief examination of the literature regarding GAS in the human pharynx will aid in evaluation of the validity of the mouse pharyngeal colonization model. For additional information, there exist a number of excellent review articles and book chapters that have focused exclusively on GAS colonization of the human pharynx (DeMuri and Wald, 2014; Martin, 2016; Wessels, 2016).

### Defining GAS-Host Interactions in the Human Pharynx

GAS is responsible for over 600 million global cases of pharyngitis annually, with rates of GAS symptomatic cases occurring in 8–40% of schoolchildren (Shaikh et al., 2010). Pharyngitis cases present clinically with abrupt onset of fever, malaise, and suppurative complications. Patients display increased serologic titers of multiple anti-GAS antibodies after infection, which can remain elevated for many months (Johnson et al., 2010). Treatment failure after antibiotic courses in symptomatic cases can lead to persistence of GAS in the human pharynx for months to years (Kaplan and Johnson, 2001; Kaplan et al., 2007).

In addition to symptomatic pharyngitis cases, asymptomatic GAS cases have been described in the scientific literature for over 75 years, with a recent meta-analysis finding that overall, 12% of healthy children showed the presence of GAS in the pharynx with no signs or symptoms of pharyngitis (Shaikh et al., 2010). Although the demarcation of subclassification within the asymptomatic cases is a contentious topic, some experts differentiate two distinct groups within the asymptomatic cases: the GAS carriers, and those with subclinical infection. A review discussing the major differences and characteristics between acute infection, subclinical infection, and carriage has been published by DeMuri and Wald (2014). However, the differentiation between these states in the human is beyond the scope of this review, so throughout the text we will solely be referring to acute infection and asymptomatic colonization, even though the asymptomatic state is multi-faceted.

Our inclusion of the discussion of asymptomatic pharyngeal colonization stems from our desire to determine whether mouse models used in the literature are closer simulations of symptomatic pharyngitis or asymptomatic colonization. Asymptomatic colonization remains an area of concern, as patients with asymptomatic GAS may have increased prevalence of disease as compared to uncolonized individuals. A longitudinal study conducted on 693 children found higher rates of neurological symptoms in children asymptomatic of pharyngeal disease when compared with children testing negative for GAS (Murphy et al., 2007), suggesting an increase in negative neuropsychiatric outcomes among groups of asymptomatically colonized individuals. Pediatric autoimmune neuropsychiatric disorders associated with streptococcal infections (PANDAS) most typically present as an onset of obsessive-compulsive behaviors (OCD) or motor or vocal tics in children following a GAS infection (Swedo et al., 1998). Additionally, another study found that rates of transmission between an individual carrying GAS and a second household member was at 9%, indicating that asymptomatically colonized individuals serve as a GAS reservoir and can contribute to the number of acute infections and post-infectious sequelae in their communities (James et al., 1960). These data show that patients with asymptomatic GAS can experience negative outcomes and therefore, determination of bacterial and host factors that play a role in pharyngeal colonization may improve our ability to identify and treat these patients.

### Bacterial Mechanisms of GAS Infection and Persistence in the Human Pharynx

Elucidating the molecular mechanisms contributing to the development of GAS pharyngitis in humans has been a concentrated area of study, and a basis for understanding bacterial factors and regulatory systems impacting virulence in the pharyngeal niche has been revealed. To start, the two-component regulatory system CovRS appears to be essential for the survival and persistence of GAS in the pharynx (Trevino et al., 2009; Wessels, 2016). CovRS controls ~15% of the genome including the synthesis of the hyaluronic acid capsule, a variety of surface proteins, and 24 virulence factors (Graham et al., 2002). Many of the downstream targets of CovRS have been investigated for their importance in pharyngitis. Inactivating mutations in the CovRS system are seen in 40% of clinical isolates from patients with toxic shock syndrome or necrotizing fasciitis but are rarely observed in pharyngitis isolates, implying there are key differences between the adaptive genetic programs in these states of infection (Ikebe et al., 2010; Shea et al., 2011).

While factors important to GAS pharyngitis have been studied for many years, there is much less known about the factors that govern the asymptomatic colonization by GAS. Intracellular survival is one potential method that the bacteria could utilize to persist in the host asymptomatically for long periods of time. Although classically considered an extracellular pathogen, LaPenta et al. first demonstrated that GAS were efficiently taken into cultured respiratory epithelial cells (LaPenta et al., 1994). Osterlund et al. later used electron microscopy and immunohistochemistry to show that human tonsillar cells isolated from patients also contained viable internalized GAS (Osterlund and Engstrand, 1997; Osterlund et al., 1997). Within cells, GAS are often observed in elongated chains, which can underrepresent the number of viable cells when homogenized tissue is plated for colony-forming units, necessitating the use of microscopy for accurate counts (O'Neill et al., 2016). Osterlund et al. (1997) examined the tonsils of 14 patients with tonsillitis, with 93% exhibiting the presence of intracellular GAS in pharyngeal epithelial cells. Eleven additional patients that had been identified as asymptomatically colonized were examined, with 73% showing GAS inside macrophages and 36% within epithelial cells. Internalization of GAS in a variety of cultured cell types (including leukocytes) is now a well-accepted phenomenon (Fischetti and Dale, 2016) and should remain an area of study when identifying factors aiding in GAS colonization of the human pharynx.

In a large-scale survey of GAS pharyngeal colonization, Peters et al. obtained throat cultures from over 10,000 Air Force cadet recruits at the beginning and end of their 6 week basic training, and saved the cultured strains from cadets both acutely and asymptomatically colonized with GAS (Peters and Gackstetter, 1998). Using four strains isolated from the asymptomatic cadets, MGAS12501-12504, Beres et al. showed that there was no common signature genetic polymorphism(s) that differentiated the asymptomatic strains from acute infection isolates. However, two of the four asymptomatic strains shared a 12 bp deletion in the promoter of a major virulence regulator of GAS, *mga* (Beres et al., 2006). Flores et al. further investigated the 12 bp deletion and found that the expression of *mga* was decreased significantly at every part of the growth phase (Flores et al., 2013). Furthermore, decreased expression of *mga* led to decreased expression of genes encoding important virulence factors such as M-protein (*emm*), C5a peptidase (*scpA*), and streptococcal collagen-like protein (*sclA*).

In additional studies, Flores et al. identified a number of other point mutations that were found in separate strains isolated from asymptomatically colonized individuals. One isolate displayed a frameshift mutation in the *hasA* gene, which decreased the production of capsule by over 100-fold (Flores et al., 2014); another contained a missense mutation in the response regulator LiaS, decreasing the expression of pilus components (Flores et al., 2015b, 2017); and a third had a polymorphism in the gene encoding streptococcal collagen-like protein A (Flores et al., 2015a). In another study, 10 unrelated individuals provided periodic throat swabs over 8 months. The genomes of the isolates were sequenced to identify mutations that emerged in individual subjects over time. Three of the 10 individuals were colonized with strains that had mutations in *hasA* or *hasB* over the course of the experiment, indicating that downregulation of capsule biosynthesis has a strong correlation with GAS persistence in the human oropharynx (Flores et al., 2014). Another of the subjects contained an isolate that reverted a premature stop codon in the *sclA* gene, restoring the encoded protein to a functional length found in the majority of clinical isolates (Flores et al., 2015a). The findings indicate that carrier strains of GAS develop mutations that cause the reduced expression of virulence genes and down-regulate proteins and polysaccharides that are possibly recognized by the host. Apart from these studies, little is known about the molecular mechanisms underlying the carrier state, and perhaps future studies from carrier isolates will help to fill the gaps alongside knowledge gained from animal models.

### Host Mechanisms Active During GAS Infection and Persistence in the Human Pharynx

Aspects of the human innate and adaptive immune response involved in interactions with GAS under culture conditions and *in vitro* settings are numerous and, and have been reviewed recently (Flores et al., 2012). However, far fewer *in vivo* studies have involved human patients to identify important immune components in the pharyngeal interaction with GAS. One such study obtained palatine tonsil tissue from patients undergoing tonsillectomy due to recurrent tonsillitis (RT) and compared them to excised tonsils from patients with tonsillar hypertrophy (Kerakawauchi et al., 1997). The tissues from RT patients displayed higher presence of GAS and significantly more cells producing IgA and IgG against M protein. These patients also had significantly increased proliferation of CD4+ T cells and production of IFN-γ, IL-2, IL-4, IL-5, and IL-6 compared to the tissues from tonsillar hypertrophy.

A recent study performed phenotypic, genotypic, and functional studies on tonsils of pediatric patients with GAS RT, comparing them to pediatric tonsils without RT (Dan et al., 2019). As patients with RT are less able to clear pharyngeal GAS, the insights gained from this study show the host immune factors necessary for clearing and responding to GAS pharyngeal infections. Histological examination of RT tonsils revealed smaller germinal centers compared to non-RT tonsils, suggesting a potential CD4+ T cell defect in RT disease. The deficiency in CD4+ T cells was confirmed by flow cytometry and shown to be in a particular subtype of CD4+ T cells—the germinal center CD4+ T follicular helper cells (GC-T_FH_ cells). Additionally, the RT patients had lower populations of GAS-specific GC-T_FH_ cells when compared to the control cohort. Because germinal center responses depend on major histocompatibility factor class II (MHC -II) antigen presentation by B cells to GC-T_FH_ cells, human leukocyte antigen (HLA)-types were compared between RT and non-RT patients to determine whether certain alleles predisposed patients to RT. One allele, HLA DQB1^*^06:02, has been previously shown to be protective against development of toxic shock syndrome, invasive GAS infection, and rheumatic heart disease (Kotb et al., 2002; Stanevicha et al., 2007). Dan et al. (2019) found that in patients with RT, the incidence of this allele was significantly less frequent than in the non-RT patients, suggesting a protective effect of DQB1^*^06:02 in RT as well as in invasive disease and rheumatic heart disease. The authors also identified two HLA alleles that were associated with increased risk of RT disease: HLA DRB1^*^01:01 and DRB1^*^07:01. Finally, the RT children were shown to have decreased antibodies to the MHC-II GAS superantigen *speA*, and the DRB1^*^01:01 and DRB1^*^07:01 were confirmed to poorly bind the superantigen when compared to the protective allele, DQB1^*^06:02.

Identification of these mechanisms shows that the immune response of the host is crucial to the persistence of GAS in the pharynx. Comparison of this data to murine immune responses seen during GAS pharyngeal colonization experiments will aid in evaluating the animal model's strength later in this review.

## The Murine Pharyngeal Colonization Model

The vast majority of animal experiments that have examined interactions between GAS and the pharynx have been conducted in a murine model, typically referred to as the pharyngeal or mucosal colonization model (Watson et al., 2016). In this model, the mice are anesthetized and a total volume ranging from 2.5 to 100 μl is placed on the nares and inhaled by the mice. The droplets generally contain 10^6^−10^8^ CFU of bacteria which become distributed throughout the nasopharynx, oropharynx, and nasal-associated lymphoid tissue (NALT), and in the instances using larger droplet volumes, bacteria are also found in the lungs. Monitoring of bacterial burden is carried out either by using calcium alginate throat swabs that are released into saline and plated onto selective media for CFU enumeration or by plating homogenized nasal tissue onto selective media.

### Early Murine Models

The use of mice as a model for streptococcal pharyngeal colonization originated when Rebecca Lancefield's group isolated a strain of GAS (B514/33) from an epizootic infection of a mouse colony (Hook et al., 1960). The strain showed high ability to colonize the mouse pharynx, with ~60% of inoculated mice remaining colonized for over a month. However, 40 years later, Yung et al. discovered that the B514 mouse-derived strain lacked a functional positive regulatory gene, *mga*, which meant that many of the virulence factors under Mga control, such as M and M-related proteins, known to be important in the development of pharyngitis, were not expressed (Yung et al., 1999). Surprisingly, when the *mga* locus in B514 was replaced with a functional copy of *mga* from an M4 strain, the incidence of colonization using the same intranasal inoculation protocol did not change despite restored production of M-related proteins (Limbago et al., 2001). Relevance of the B514 mouse strain to human disease remains uncertain, and thus, studies have transitioned to inoculating mice with clinical isolates obtained from human patients. Here we describe contemporary murine pharyngeal colonization studies that have been used to identify important GAS genetic regulatory elements for pharyngeal colonization, to pinpoint elements of the murine immune system that interact with GAS, and to test vaccine candidates for mucosal immunity against pharyngeal infection. Following a summary of the results, principal conclusions will be compared to the previously-discussed findings in human patients.

### GAS Structural and Regulatory Components Found to Impact Pharyngeal Colonization

Several genes, including those which encode surface proteins, channels, capsule biosynthesis, and regulatory systems, have been shown to affect GAS pharyngeal colonization. As the CovRS two-component system regulates a large portion of the GAS genome, Alam et al compared wild type and a Δ*covR/S* mutant for their abilities to maintain colonization of female FVB/n mice over several days. The wild type was detectably shed for longer periods of time than the mutant, confirming that presence of CovRS aids in colonization of the nasopharynx (Alam et al., 2013). These data are consistent with the finding that the CovRS two-component system is rarely mutated in human pharyngeal isolates, unlike what is seen in isolates from invasive infections where inactivating mutations are isolated frequently (Liang et al., 2013; Galloway-Pena et al., 2018). The results support the notion that CovRS provides transcriptional adaptation to the pharyngeal niche.

The expression pattern of the cysteine protease SpeB is typically inversely related to CovR-repressed genes and its impact on GAS survival during skin and soft tissue infections is well-documented (Johansson et al., 2010; Mayfield et al., 2014; Brouwer et al., 2018). The importance of SpeB in pharyngeal colonization, along with the RopB-SIP quorum sensing system that regulates *speB* expression, was recently tested (Makthal et al., 2018). Deletions of *speB, ropB*, or the small open reading frame encoding the SIP pheromone each led to decreased colonization of the mouse oropharynx when examined via cultured throat swabs over a period of 7 days. These exciting results not only demonstrate that SpeB plays a role in pharyngeal colonization but indicate that cell-cell signaling is a vital component of the ability of GAS to persist in the murine oropharynx.

For any microbe to colonize its host, acquisition of nutrients and energy is essential. In the oropharynx, polysaccharides found in saliva and on mucosal surfaces could provide carbon sources for growth. The MalE and MalT proteins aid in the import of salivary polysaccharide catabolites during growth in human saliva (Shelburne et al., 2006). MalE, a maltodextrin binding protein located on the GAS surface, was seen to be recognized as a common GAS antigen by pooled human immunoglobulin in 20 out of 20 tested GAS isolates (Reglinski et al., 2015). Shelburne et al. found that a Δ*malE* isogenic mutant had decreased growth in human saliva and was deficient in colonization of the murine pharynx (Shelburne et al., 2006). In a follow-up study, Δ*malT* was also attenuated its ability to colonize of the murine pharynx, implying that maltodextrin obtained from host salivary polysaccharide is an important carbon source for the bacteria in the pharyngeal colonization model (Shelburne et al., 2008).

As described above in human studies, Flores et al. have undertaken the sole studies of sequencing and identifying mutations in isolates from asymptomatic human GAS carriers (Flores et al., 2013, 2014, 2015a,b, 2017). The genetic variants identified provide a means to test whether the asymptomatic phenotype seen in colonized humans translates to the mouse pharyngeal colonization model. In one of these surveys, throat swabs were serially collected from individuals at times of acute infection and later asymptomatic colonization with the intention of comparing genetic differences in strain isolates (Flores et al., 2015b). From one individual in this study, strain MGAS23412 was obtained during an episode of pharyngitis, and a second throat swab taken 63 days later during asymptomatic colonization, producing strain MGAS23431. Three genetic changes were found in the latter, asymptomatic strain: one in *liaS*, one in *covR*, and one in a subunit of an ATPase (*spyM3_0499*). LiaS, also called YvqE, has been studied sparingly in GAS. It is a sensor kinase in a regulatory system which has shown importance in growth in acidic media, survival in whole blood, and subcutaneous murine infection (Ichikawa et al., 2011). The mutation identified in MGAS23431 (*liaS*^*R135G*^) was generated in another strain, MGAS10870. The MGAS10870 *liaS* mutant showed increased colonization in the murine pharyngeal model when compared to its derivative strain, in which *liaS* was restored to wild type. Thus, it was concluded that LiaS was a contributing factor to asymptomatic adaptation in the human host. Contributions provided by mutations in *covR* and the ATPase were not assessed. Subsequent work showed that *liaS* controls transcription of the *tee3* gene, which encodes a structural component of the pilus, and that deletion of *tee3* also significantly decreases oropharyngeal colonization in the mouse (Flores et al., 2017).

In a separate study, an isolate from an asymptomatic patient contained a mutation in the *sclA* gene encoding streptococcal collagen-like protein (Flores et al., 2015a). The mutation was a 153-bp in-frame deletion that eliminated an internal stop codon commonly seen in *sclA* of invasive M3 serotype strains of GAS. The deletion allows for translation of a fully functional surface adhesin. The intact-*sclA* variant strain displayed decreased virulence in a necrotizing fasciitis model and decreased growth in human blood. Generation of the mutant allele of *sclA* in an invasive M3 strain showed slightly lower rates of colonization of the mouse nasopharynx, and with lower bacterial yields, than the isogenic invasive parent strain.

Another asymptomatic isolate, strain MGAS12503, yielded a genetic polymorphism in the *hasA* gene (Peters and Gackstetter, 1998; Flores et al., 2014). The *hasABC* operon is responsible for the synthesis of the hyaluronic acid capsule that covers the surface of GAS cells and is important in evading phagocytosis. The mutant strain was found to be far less virulent in models of necrotizing fasciitis and survival in human blood, but it was also less adept at colonizing the oropharynx from the strain in which *hasA* was repaired by designed genetic recombination. The original mutant MGAS12503 colonized none of the mice while the complemented MGAS12503*hasA*^wt^ colonized ~70% of the mice until 14 days (Flores et al., 2014).

Summarized findings of all the mouse pharyngeal colonization experiments conducted by Flores et al with the asymptomatic human isolates can be found in Table 1. Both the *sclA* and *hasA* variants taken from asymptomatic patients were less capable of colonizing the mouse pharynx, whereas the *liaS/tee3* mutations displayed increased ability to colonize. The incongruency between asymptomatic isolates from humans and their behavior during murine pharyngeal colonization sheds light on possible limitations of this model, which will be further discussed in the final section.

**Table 1:** Comparison of asymptomatic isolates and their behavior in various *in vitro* and *in vivo* models.

**Mutation in asymptomatic human isolate**	**Mutation effect**	**Effect on murine pharyngeal colonization**	**Effect on blood survival/neutrophil survival (*ex vivo* assays)**	**Effect on virulence of murine necrotizing fasciitis model**	**Source**
*Mga*	Downregulation	*Not studied*	Decreased	Decreased	Flores et al., 2013
*hasAB*	Downregulation	Decreased	Decreased	Decreased	Flores et al., 2014
*sclA*	Reversion to full-length protein	Decreased	Decreased	Decreased	Flores et al., 2015a
*liaS*	Downregulation	Increased	Decreased	Decreased	Flores et al., 2015b, 2017
*tee3*	Upregulation	Increased	*Not studied*	Decreased	Flores et al., 2017

### Elements of the Murine Immune System Important in Pharyngeal Colonization

**Figure 1:**
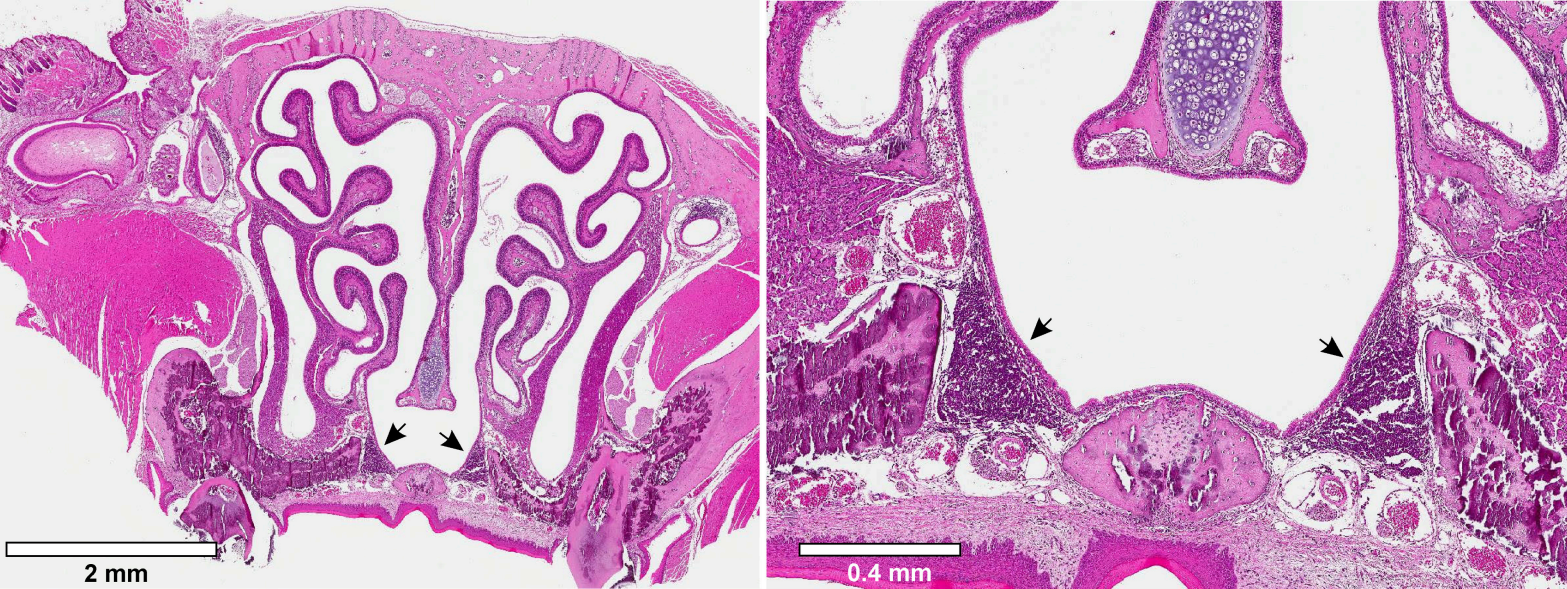
Murine Nasal-associated Lymphoid Tissue (NALT). Transverse cross-sections through the nasal cavity approximately were obtained approximately half-way through the nasal cavity. The tissue was fixed and stained with hematoxylin and eosin. Arrowheads indicate the bilateral murine NALT.

As discussed previously, the tonsils play an important part in the pathogenesis and carriage of GAS in the human pharynx. Although mice lack tonsils, they possess a nasal-associated lymphoid tissue (NALT) that is believed to behave analogously to the tonsils. The NALT is present in all rodents and consists of bilateral lymphoid structures situated dorsal to the soft palate (Figure 1). The NALT behaves as a mucosal inductive site, mediating mucosal immunity via uptake and response to antigens (Csencsits et al., 1999). The periphery of the NALT consists of an epithelial layer with specialized M cells that mediate antigen uptake (van der Ven and Sminia, 1993). Within the NALT are zones of B cells and T cells that respond to antigens and are arranged in a manner that mirrors the architecture of Peyer's patches in the gut (Asanuma et al., 1997). The site has also been found to be crucial for immunity to a number of viruses introduced intranasally (Tamura et al., 1998; Zuercher et al., 2002), and is an important part of the murine immune response against colonization of the oronasopharynx.

Examination of the NALT during the GAS intranasal inoculation model was studied extensively by Pat Cleary's group. Building on structural similarities of NALT to gut Peyer's patches and the notion that certain pathogens like *Salmonella* and *Shigella* breach epithelial tissues through these sites (Sansonetti and Phalipon, 1999), Park et al. found that M cells of the murine NALT were also crucial ports of entry for GAS, and they demonstrated that M cells aided in transfer of the bacteria deeper into the NALT lymphoid tissue (Park et al., 2003). Infecting the murine nasopharynx with a bioluminescent reporter-expressing strain indicated that the anatomical distribution of the bacteria during infection was concentrated in the nasal area. Dissection of the region showed that the NALT and the airway mucosa were heavily colonized. A time-course revealed that an initial depletion and subsequent rebound of bacterial viability occurred soon after introduction of bacteria to the nasal passage. Immediately following inoculation, the NALT contained 10^9^ CFU, whereas 2 h later only 10^3^ CFU were recovered. A rebound in bacterial numbers (reaching 10^7^ CFU) was seen by 24 h, and over the next 7 days, the numbers diminished again until bacteria were undetectable in the NALT on day 8. Cell suspensions from infected NALT showed that the bacteria were living both intracellularly and extracellularly in the lymphatic milieu, with 1–10% of the total bacteria living intracellularly.

Cleary's group continued to probe the importance of interactions between the murine immune system and bacteria. Intranasal infection of GAS in immunocompetent mice induced an influx of neutrophils into the NALT at 24 h, which declined by 48 h. Intranasal infection was also shown to activate CD4+ helper T cells in the NALT more robustly than in draining lymph nodes and the spleen, where there was little to no activation (Park et al., 2004). In order to enhance physiological relevance to the human immune system and allow for more detailed characterization, John McCormick's group developed a model using humanized mice (Kasper et al., 2014). The mice were engineered to express human MHC-II which, as previously discussed, is the target of GAS superantigens. Multiple HLA alleles were used. The HLA-DR4 mice contained ~100-fold CFUs of S. pyogenes compared to wild-type B6 mice while HLA-DQ8, or mice expressing both HLA-DR4 and HLA-DQ8 contained ~10,000-fold more CFUs than wild-type in their nares. The increase in colonizing CFU was dependent on the expression of superantigens by the bacteria, and was reverted back to levels seen in wild-type mice when SpeA was deleted in MGAS8232. Additionally, expression of superantigens by the bacteria helped to mount a strong inflammatory response that could be used as a vaccine to inhibit nasopharyngeal colonization.

Using an engineered strain of GAS expressing an antigenic epitope (2 W) fused to the M1 protein, Dileepan et al. were able to further characterize the CD4+ helper T cell response using similar transgenic mice expressing the human I-Ab MHC-II molecule (Dileepan et al., 2011). Following intranasal inoculation with 2W-GAS, CD4+ T cells were found in the NALT and lymphokine assays performed with heat-killed 2W-GAS showed that these T cells produced IL-17A. CD4+ cells isolated from mice infected intravenously or subcutaneously with 2W-GAS showed a Th17/Th1 ratio that was 40 times less than the intranasally infected mice, indicating that the Th17 response is dependent on the intranasal route of infection. Other experiments showed that adoptive transfer of CD4+ T cells from immunized IL-17A(+)/(+) mice, but not cells from IL-17A(-)/(-) mice, allowed naïve mice to clear intranasal GAS (Wang et al., 2010), further confirming the importance of the IL-17 response. The IL-17A+ T cells are also dependent on IL-6, as IL-6(-)/(-) mice failed to develop a Th17 response and lost the ability to clear GAS infection (Dileepan et al., 2011).

When the mice were recurrently infected intranasally with the 2W-engineered bacteria, the antigen specific population of CD4+ T cells in the NALT was shifted toward an IL-17A+ IFN-γ+ double-positive phenotype. Infection of IFN-γ(-)/(-) mice showed an even higher influx of neutrophils during GAS infection. Additionally, infection of IFN-γ(-)/(-) mice with GAS resulted in local dissemination of bacteria to draining lymph nodes, while in immunocompetent mice the bacteria were primarily confined in the NALT. This suggests that IFN-γ is required to prevent dissemination of GAS to draining lymph nodes (Hyland et al., 2009). In summary, the humanized MHC-II murine model showed that IFN-γ prevents dissemination of GAS to draining lymph nodes while IL-17A aids in the clearance of the bacteria from the lymphatic system.

### Utilization of Pharyngeal Model to Evaluate Mucosal Immunity in Vaccine Studies

The murine pharyngeal model has been used to evaluate mucosal immunity in vaccine studies using various GAS surface targets. The most studied target has been the M protein, either versions of the entire mature protein or peptide fragments conjugated to diphtheria or cholera toxins. Bessen and Fischetti conjugated a conserved-domain epitope of M6 to the cholera toxin B subunit (CTB) and administered the conjugate intranasally to 4–5 week old CD1 mice (Bessen and Fischetti, 1988). Ten days following vaccination and boost, animal-passaged GAS strain S43/192 was intranasally inoculated into vaccinated mice (2.5 × 10^8^ CFU). Mouse throats were sampled for GAS with calcium alginate swabs until day 15. Animals that received the conjugate vaccine had significantly lower colonization of the pharynx compared to the control group, which was immunized only with CTB. Evaluation of the antibody response to immunization showed that vaccinated mice had high titers of both IgG and salivary IgA against the M-protein peptides, confirming that mucosal immunity was stimulated. Olive et al. conducted a similar study utilizing a different vaccine formulation and also found significantly lower colonization numbers in vaccinated mice when compared to the PBS control (Olive et al., 2006). Another study utilized a probiotic strain of *Lactobacillus gasseri* engineered to express M6 protein for intranasal immunization (Mansour and Abdelaziz, 2016). Oral immunization with *L. gasseri* and subsequent intranasal challenge with GAS led to a 10% colonization rate via throat swabbing, whereas 45% of the non-immunized mice were colonized.

The N-terminal domain of M is highly variable and accounts for serotype designations; identification of vaccine targets that are more highly conserved among all strains would likely be more efficacious toward a majority of serotypes. Thus, in addition to M protein, other surface proteins have been identified as targets for active immunization of the nasal mucosa. The hemoprotein receptor Shr and the fibronectin-binding protein SfbI have each been utilized in intranasal vaccination, and both mounted high IgG and secretory IgA titers, although mice were not assessed using the GAS colonization model (Huang et al., 2011). Park et al. showed that deletion of the C5a peptidase, *scpA*, diminished the ability of GAS to persist in the NALT (Park and Cleary, 2005). Within the first 24 h, CFU counts in mice colonized with the *scpA* mutant were 5-fold lower than wild-type, and within 72 h the counts were 600-fold lower, providing a strong basis to use ScpA as a vaccine target. Mice were intranasally inoculated with recombinant ScpA, with or without cholera toxin (CTX) adjuvant, and later inoculated with a bioluminescent strain of GAS. Mice receiving ScpA + CTX displayed very little bioluminescence in the nose by 24 h, while mice receiving solely ScpA without CTX showed no difference from unvaccinated mice. CFU counts of the three mouse groups mirrored the bioluminescence data. Serum IgG and salivary IgA responses against ScpA were induced in both the ScpA+CTX and ScpA vaccinated groups, although the ScpA+CTX showed slightly higher titers.

Fan et al. identified Sortase A as a target for mucosal immunity (Fan et al., 2014). Intranasal vaccination with Sortase A, a conserved cell surface protein, reduced colonization of the NALT. Mice were vaccinated with live GAS bacteria of serotype M1 three times and then intranasally inoculated with one of three serotypes: M1, M49, or M28. Bacterial counts from the NALT were significantly lower from the control in all three infections, indicating cross-serotype protection by the M1 strain and prompting the authors to search for another conserved protein contributing to the immune response. SrtA+CTB was intranasally inoculated into mice, diminishing the ability of four different GAS serotypes to colonize the NALT. Building on previous work examining the Th17 response, Fan et al. showed that the proportion of CD4+ IL-17+ cells increased in the NALT following vaccination with SrtA-CTB. RT-PCR analysis of the NALT showed increased *Il17a* and *Ifn*γ mRNA in the mice immunized with SrtA-CTB, indicating that a Th17 response had mounted solely by the vaccination. Adoptive transfer of CD4+ IL-17+ T cells from immunized mice to naïve mice diminished the bacterial counts in the NALT compared to the control, indicating that the immune response was antibody-independent, and therefore B-cell independent. B-cell deficient mice were immunized with SrtA-CTB and remained able to clear bacteria from the NALT as efficiently as wild-type mice. Zeppa et al. also examined the T-cell response to oropharyngeal GAS after finding that passive immunization with superantigen-neutralizing antibodies protected mice from nasopharyngeal colonization (Zeppa et al., 2017). Vaccination with superantigens decreased the responsiveness of Vβ8+ T cells, leading to decreased bacterial counts by throat swabbing.

### Murine Model Optimization

Many of the aforementioned studies diverge in their use of mouse strain, mouse age, mouse sex, inoculum volume, and bacterial strain. Shiranee Sriskandan's group took on an effort to systematically optimize the mouse pharyngeal model, experimenting with various mouse lines, mouse sex, mouse ages, and streptococcal strains (Alam et al., 2013). Bioluminescent *Citrobacter rodentium* were first utilized to determine the optimal intranasal droplet size required to distribute bacteria throughout the nasopharynx but avoid seeding bacteria into the lungs, which could trigger a systemic infection. Based on luminescence imaging, the optimal inoculation volume at which there was no seeding of bacteria in the lungs was determined to be 5 μl. The study utilized five different mouse strains (CD1, Balb/c, FVB/n, C57BL/6, A/J) and clinical GAS isolates of M-types 1, 2, 75, and 81, ultimately concluding that FVB/n mice were colonized best with the M75 type clinical isolate. Further experimentation showed that younger mice (5 vs. 10 weeks) sustained longer colonization, and that in general, males were colonized better than females, although at 5 weeks the difference was not as stark. Instead of counting CFU from throat swabs, a new protocol was developed to measure the shedding of GAS from the nares of the mice by tapping their noses 10 times on an agar plate and enumerating CFU, eliminating the need for anesthesia after inoculation. The CFU shed by the mice was directly correlated to the number of CFU found in the dissected and homogenized nasopharynx. As previously discussed, the optimized infection model was then used to evaluate the importance of the CovR/S system on GAS nasopharyngeal colonization (Alam et al., 2013).

In an effort to program the bacteria to into mimicking a less virulent state, closer to bacteria in a state of asymptomatic colonization, Marks *et al*. altered the model by pre-culturing GAS on paraformaldehyde-fixed keratinocytes so that the inoculum would be grown in an environment mimicking colonization—a biofilm on a layer of epithelial cells—prior to application to the mouse nasopharynx (Marks et al., 2014). Bacteria grown in biofilms showed decreased virulence in an intraperitoneal sepsis model—no mice in the biofilm-infected group were found dead, while 30% of the mice in the planktonic-culture infection group died. After 24 h, blood was taken from the mice and enumerated—the CFU counts were a 100 to a million-fold higher in the planktonic-infected mice, depending on the GAS strain used. Additionally, isolation of RNA showed that the expression of virulence factors such as *hasABC, emm, speB, sagA*, and *slo* decreased in the biofilm-grown bacteria as compared to planktonic bacteria. Intranasal inoculation of the biofilm-grown bacteria led to significantly higher counts of bacteria in the nasopharynx and NALT, with bacteria in the NALT persisting at high CFU at a 9-day timepoint. Furthermore, colonization of the NALT allowed for natural transformation of GAS with a spectinomycin resistance cassette provided intranasally, the first time that natural transformation of GAS was observed *in vivo*. The transformation of GAS lends confidence in the model, as there is clear evidence of horizontally transferred genetic material acquired by clinical isolates over subsequent isolations (Bessen and Hollingshead, 1994; Whatmore and Kehoe, 1994; Sumby et al., 2005; Turner et al., 2015). Marks et al. provide a new model for asymptomatic carriage in the mouse, inoculating with bacteria more closely resembling a carrier state rather than a virulent, broth-grown inoculum. A study comparing immune features between this modified mouse model and clinical knowledge of human asymptomatic colonization would further strengthen the applicability of biofilm-grown GAS to a carriage model.

### Murine Vaginal Colonization Model

Although our discussion has focused on the murine pharyngeal colonization model, there is an additional murine mucosal colonization model that should be mentioned. The vaginal colonization model has been developed to allow for prolonged asymptomatic mucosal carriage, using the vaginal tract as the target (Watson et al., 2013). The mice are pretreated with estradiol to synchronize estral cycles after which 20 μl of GAS is inoculated into the vaginal vault using a pipette. Monitoring of the mice for GAS colonization is conducted by washing the vaginal vaults of sedated mice with 50 μl PBS and plating on selective medium. This model has seen colonization of the vaginal mucosa persist for as long as 1 month. Analysis of the murine immune system yielded similar findings to those found in the murine NALT. Successful colonization was achieved with multiple GAS isolates whose vaginal colonization led to a neutrophilic infiltrate in the vaginal vault and higher levels of IL-6 and IL-17A (Carey et al., 2016). Carey et al. hypothesized that the IL-17A was aiding in clearance of the bacteria from the vagina, and indeed IL-17A(-)/(-) mice had higher colonization counts and the vaults showed less infiltration of neutrophils and monocytes. There are many studies that show that the same immune processes are applicable for both the upper respiratory tract and the vagina (Bergquist et al., 1997; Garulli et al., 2007), and the importance of the Th17 response for bacterial clearance in both the nasal and vaginal mucosal models supports the use of the vaginal model as a complementary alternative to the intranasal model.

## Evaluation of the Validity of the Mouse as a Model for Human Pharyngeal Colonization

Unsurprisingly, it is difficult to adapt a human-restricted bacterium to a murine model, but nonetheless there have been many findings that are congruent between the mouse and human. A careful comparison of findings in murine and human pharyngeal colonization can be found in Tables 1, 2.

**Table 2:** Comparison of coupled findings in human and murine pharyngeal colonization studies, with supporting references.

**Findings in human pharyngeal colonization**	**References for human data**	**Murine pharyngeal colonization**	**References for murine data**
CovRS mutants are rarely isolated from human pharynx but often from skin/tissue, implying potential importance of the transcriptional regulator in the pharynx.	Ikebe et al., 2010; Shea et al., 2011	CovRS mutants were shed from the nasopharynx for significantly less time than the wild-type bacteria.	Alam et al., 2013
Proliferation of CD4+ T cells in the tonsils with production of IFN-γ, IL-2, IL-4, IL-5, IL-6 (Note: IL-17 was not evaluated).	Kerakawauchi et al., 1997	Proliferation of CD4+ T cells in the NALT with production of IFN-γ, IL-6, and IL-17 (Note: IL-2 and IL-4 were not evaluated).	Park et al., 2004; Dileepan et al., 2011
Intracellular bacteria are present in tonsillar cells (epithelial, macrophages, neutrophils) of both acutely infected and asymptomatically colonized patients.	Osterlund and Engstrand, 1997; Osterlund et al., 1997	1–10% of total bacteria in the NALT were living intracellularly.	Park et al., 2003
Certain MHC-class II alleles are associated with recurrent tonsillitis.	Dan et al., 2019	Replacement of the mouse MHC-II with human MHC-II allows for increased colonization of mice.	Kasper et al., 2014; Zeppa et al., 2017

Most of the studies identifying bacterial factors that aid in colonization of both the human and murine pharynx have been undertaken by James Musser's group. Table 1 provides a comparison of the way in which bacterial mutants isolated from asymptomatic human patients behave when subjected to the murine pharyngeal colonization model. Mutants isolated from asymptomatic human patients sometimes colonized the mouse pharynx better than when the gene was restored to wild-type (*liaS*), and sometimes colonized worse when the gene was restored to wild-type (*sclA, hasAB*) (Flores et al., 2014, 2015a,b). However, all of the asymptomatic isolates showed decreased survival in blood and decreased virulence in a murine model of necrotizing fasciitis, suggesting that asymptomatically colonizing isolates are less virulent. Flores et al. postulate that during the course of colonization, certain mutations arise in pharyngeal GAS bacteria that allow for persistence of that strain for long periods of time in an asymptomatic host. Thus, a strain isolated from an asymptomatic human, either with or without a previous instance of pharyngitis, is likely genetically divergent from the bacterium that initially colonized that human. To expect mutant isolates to be able to actively colonize the murine nasopharynx better than a wild-type strain is not necessarily feasible, as these strains evolved to persist in an environment where they had already taken hold, not to be transmitted to a new environment and colonize that new environment better than wild-type bacteria. In fact, high expression of capsule by GAS has been shown to be necessary for evading initial clearance in the murine model (Wessels and Bronze, 1994). If the expectation would be that isolates from asymptomatically colonized humans would colonize well in a model of pharyngeal colonization, then cumulatively, these data show that the murine pharyngeal model is not optimal for testing isolates from asymptomatic patients.

Table 2 couples much of the other data discussed in the human and murine sections of this article. Identification of intracellular bacteria was seen in the tonsillar cells of patients with pharyngitis and asymptomatic GAS (Osterlund and Engstrand, 1997). This finding was also seen in the NALT of inbred mice, with 1–10% of the total bacteria in the NALT surviving intracellularly (Park et al., 2003). In humans, strains are often found with inactivating CovRS mutations in patients with invasive infections but very rarely in patients with pharyngitis or asymptomatic colonization, implying importance in the pharyngeal niche (Ikebe et al., 2010; Shea et al., 2011). Inactivating mutations in CovRS also reduced the ability of GAS to colonize the mouse nasopharynx (Alam et al., 2013).

On the host side, human patients with pharyngitis were reported to have increased CD4+ T cell populations in their tonsils with increased production of IL-2, IL-4, IL-5, IL-6, and IFN-γ (Kerakawauchi et al., 1997). In the NALTs of inbred mice, CD4+ T cells were similarly found to proliferate upon infection with GAS, producing IFN-γ, IL-6, and IL-17 (Park et al., 2004; Dileepan et al., 2011). To our knowledge, there are no published investigations of the production of IL-17 and subsequent Th17/Th1 ratio in the tonsils of GAS-infected humans. As the Th17 cells have been shown to be important for murine mucosal immunity to GAS vaccine candidates, as well as clearance GAS from the nasopharynx (Dileepan et al., 2011), the examination of the IL-17 response in human tonsils is an important future area of investigation.

Recently, Dan et al. (2019) reported on the immunopathology underlying pediatric recurrent tonsillitis. The MHC-II molecule was the leading factor for response to the bacterium and avoidance of recurrent infection. Certain HLA types were found to be protective of or accounted for increased susceptibility to RT, emphasizing the importance of minute structural differences in MHC-II molecules when the immune system encounters GAS. With this new knowledge in hand, it is not surprising that mice with humanized MHC-II were colonized much differently than wild-type mice (Kasper et al., 2014). Different HLA alleles in the humanized mice resulted in different levels of colonization when compared to one another and to the wild-type mice. Mounting responses against SpeA was found to be crucial to the development of immunity against nasopharyngeal colonization. However, the recent findings by Dan et al. (2019) show that the precise HLA allele that is utilized in humanized mice is important and cannot be overlooked. The utilization of MHC-II humanized mice appears to provide a valuable tool for examination of the host response against intranasal inoculation with bacteria, as Dileepan et al. (2011) was able to use similar mice to identify the importance of the Th17 response.

One last question to ask is whether the murine mucosal colonization model is most representative of acute infection or asymptomatic colonization in the human. Whilst reviewing relevant literature for this review, we have been grappling with the answer to this question. As mice do not develop symptoms similar to human pharyngitis, it is difficult to tell from merely examining the mice. The only method that would clarify this question is to understand the difference between the immune response in human pharyngitis and asymptomatic colonization and compare this to the mouse findings. Unfortunately, there is little work done on the immunological differences between patients with pharyngitis and asymptomatic colonization, so the answer to this question remains unclear.

In conclusion, the mouse pharyngeal colonization model is an affordable and accessible experimental system that allows a broader group of research groups to test the importance of their findings in an *in vivo* model. There is a great deal of overlap between the bacterial and immune factors found to be important in human and murine pharyngeal colonization. Although MHC-II humanized mice could prove useful in answering certain questions, the use of inbred mouse lines appears to be sufficient for evaluation of most questions, both related to bacterial factors and the host immune response. However, when promising treatments or vaccines against GAS are identified, non-human primate models remain the gold standard and should be utilized to validate all results.

## Author Contributions

AG conducted most of the literature review and initial writing of the text. MF edited and contributed to the expansion of several sections of the review.

### Conflict of Interest Statement

The authors declare that the research was conducted in the absence of any commercial or financial relationships that could be construed as a potential conflict of interest.
